# Medizin für Menschen mit Behinderungen: Besonderheiten in der unfallchirurgischen Versorgung von Extremitätenverletzungen bei Menschen mit schwerer geistiger und körperlicher Behinderung

**DOI:** 10.1007/s00113-021-01105-4

**Published:** 2021-11-09

**Authors:** Niklas Grüneweller, Dirk Wähnert, Nathalie Schillians, Adrian Komadinic, Thomas Vordemvenne

**Affiliations:** 1 Evangelisches Klinikum Bethel, Klinik für Unfallchirurgie und Orthopädie, Universitätsklinikum OWL der Universität Bielefeld, Campus Bielefeld-Bethel, Burgsteig 13, 33617 Bielefeld, Deutschland; 2 Krankenhaus Mara Bethel, Klinik für Chirurgie des Zentrums für Behindertenmedizin, Universitätsklinikum OWL der Universität Bielefeld, Campus Bielefeld-Bethel, Maraweg 21, 33617 Bielefeld, Deutschland

**Keywords:** Geistige Behinderung, Körperliche Behinderung, Konservative Therapie, Operative Therapie, Extremitätenverletzung, Mental disability, Physical disability, Conservative treatment, Operative tratment, Extremity injury

## Abstract

Die von Bodelschwinghschen Stiftungen Bethel blicken auf eine über 150-jährige Tradition in der Behandlung von Menschen mit Behinderungen. Das traumatologische Patient*innenkollektiv ist dabei regelmäßig durch schwerste geistige und körperliche Entwicklungsstörungen und (Mehrfach‑)Behinderungen, mit und ohne Verhaltensstörungen, und internistische Begleiterkrankungen charakterisiert. Diese besondere Kombination erfordert ärztlich wie pflegerisch eine Therapie und Indikationsstellung, welche in allen Behandlungsschritten spezifisch abgewogen und angepasst werden muss. Behandlungsrichtlinien oder Empfehlungen zu diesem Patientenkollektiv existieren in der Literatur nicht. Des Weiteren kann die Behandlung von Frakturen bei Menschen mit Behinderungen nicht immer nach etablierten Konzepten erfolgen. Aufgrund einer hohen postoperativen Komplikationsrate kommt der konservativen Therapie eine entscheidende Rolle zu. Die Entscheidung zur operativen Therapie muss interdisziplinär und individuell unter Berücksichtigung sämtlicher Faktoren getroffen werden. Spezielles Augenmerk muss dabei auf das zu wählende Verfahren (Stabilität, funktionelle Bedürfnisse) gerichtet werden.

## Abstract (en)

The v. Bodelschwingh Foundation Bethel can look back on a tradition of more than 150 years in the treatment of people with disabilities. The trauma patient collective is regularly characterized by the most severe mental and physical developmental disorders and (multiple) disabilities, with and without behavioral disorders and concomitant internal diseases. This special combination requires treatment and indication assessment both medically and in nursing, which must be specifically weighed up and adapted in all treatment steps. Treatment guidelines or recommendations for this patient group do not exist in the literature. Furthermore, the treatment of fractures in people with disabilities cannot always follow established concepts. Due to a high postoperative complication rate, conservative treatment plays a crucial role. The decision for surgical treatment must be made on an interdisciplinary and individual basis, taking all factors into account. Special attention must be paid to the procedure to be chosen (stability, functional needs).

## Einleitung

Die von Bodelschwinghschen Stiftungen Bethel blicken auf eine über 150-jährige Tradition in der Behandlung von Menschen mit Behinderungen. Im interdisziplinären Therapieansatz erfolgt die stationäre Versorgung dieser vulnerablen Patientengruppe in dem eigenständigen Fachkrankenhaus für Behindertenmedizin Mara, welches in enger Zusammenarbeit mit dem Evangelischen Klinikum Bethel steht. Die Klinik für Unfallchirurgie und Orthopädie stellt dabei, neben der regulären Versorgung eines überregionalen Traumazentrums und SAV(Schwerverletztenverfahren)-Hauses, in beiden Standorten die traumatologische wie auch orthopädische Versorgung von Menschen mit Behinderung sicher.

**Figure 1:**
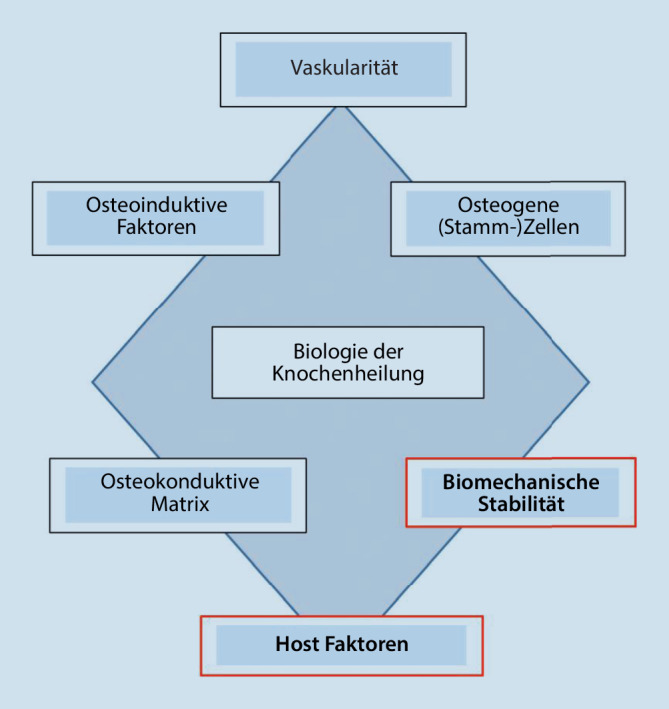

Das traumatologische Patientenkollektiv ist regelmäßig charakterisiert durch schwerste geistige und körperliche Entwicklungsstörungen und (Mehrfach‑)Behinderungen, mit und ohne Verhaltensstörungen, sowie komplizierende Epilepsien und internistische Begleiterkrankungen. Diese besondere Kombination aus einer unfallchirurgischen Verletzung und Verhaltensstörungen mit Incompliance sowie komplexen syndromalen Grunderkrankungen erfordert ärztlich wie pflegerisch eine Therapie und Indikationsstellung, welche spezifisch in allen Behandlungsschritten bis hin zur Rehabilitation abgewogen und angepasst werden muss. Behandlungsrichtlinien oder Empfehlungen zu diesem Patientengut existieren in der Literatur nicht. Es bietet sich an, das von Giannoudis et al. veröffentlichte Diamantkonzept zu betrachten; in diesem werden die wesentlichen Bausteine der Frakturheilung zusammengefasst (Abb. 1; [1, 4]). In der hier betrachteten Patientengruppe sind 2 dieser Faktoren von überproportional großer Bedeutung: die Einflüsse der Host-Faktoren und die biomechanische Stabilität. Hierbei ist zu betonen, dass sich diese Faktoren in der Therapieentscheidung nicht immer gegenseitig unterstützen. Vielmehr kann die Gewichtung der Host-Faktoren in der Behandlung von Menschen mit Behinderungen derart bestimmend sein, dass bewusst zulasten der biomechanischen Stabilität ein alternativer Therapieweg in Kauf genommen werden muss.

Im Gegensatz dazu verlangen die Host-Faktoren in anderen Fällen dieses Patientenkollektives ein Osteosyntheseverfahren mit einer unüblichen, erhöhten primären biomechanischen Stabilität (z. B. primäre Doppelplattenosteosynthese).

Die im Folgenden dargestellten Aspekte der Behandlung spiegeln diese Komplexität wider und sollen dazu dienen, die Besonderheiten aufzuzeigen und die Entscheidungsfelder einzugrenzen.

## Besonderheiten der Medizin für Menschen mit Behinderungen

Für alle Abbildungen, die eine Identifikation der Person zulassen, liegen schriftliche Einwilligung zur Veröffentlichung vor.

### Spezifische Patient*innencharakteristika (Host-Faktoren)

Die versorgten Patient*innen weisen geistige, körperliche und kombinierte Behinderungen auf. Dementsprechend finden sich unterschiedlichste und spezifische Bedürfnisse an die Funktion, die es in der Therapieentscheidung zu berücksichtigen gilt.

*Geistige Behinderungen* in unterschiedlichsten Ausprägungen von freundlich zugewandt und suffizient verbal kommunikationsfähig über teilnahmslos bis hin zu hochgradig auto- und fremdaggressiv, ohne Fähigkeit der verbalen Kommunikation, sind somit zu adressieren.

Die Anamnese, als zentrales Element der Informationsgewinnung und Therapiesteuerung, ist im besagten Patient*innenkollektiv meist deutlich eingeschränkt, sodass Angaben über Zeitpunkt und Art der Entstehung einer Verletzung, aber auch über Mobilität, Ansprüche und Compliance nicht direkt vom Patienten zu erhalten sind. Hier ist das Hinzuziehen von Betreuern und Pflegenden essenziell, um das Versorgungsbild vor dem Trauma zu eruieren. Regelmäßige interdisziplinäre Teamkonferenzen helfen, die Konzepte zeitnah anzupassen.

Komplizierend ist, dass Schmerz in diesem Patient*innenkollektiv (je nach zugrunde liegender Erkrankung) in anderer Ausprägung wahrgenommen oder präsentiert werden kann [10]. Dieses kann zum einen zu einer ungewöhnlichen Verletzungsschwere bzw. zu ungewöhnlichen Verletzungsmustern führen. Zum anderen sind protrahierte Verläufe und verzögerte Diagnosestellungen möglich (Abb. 4).

**Figure 2:**
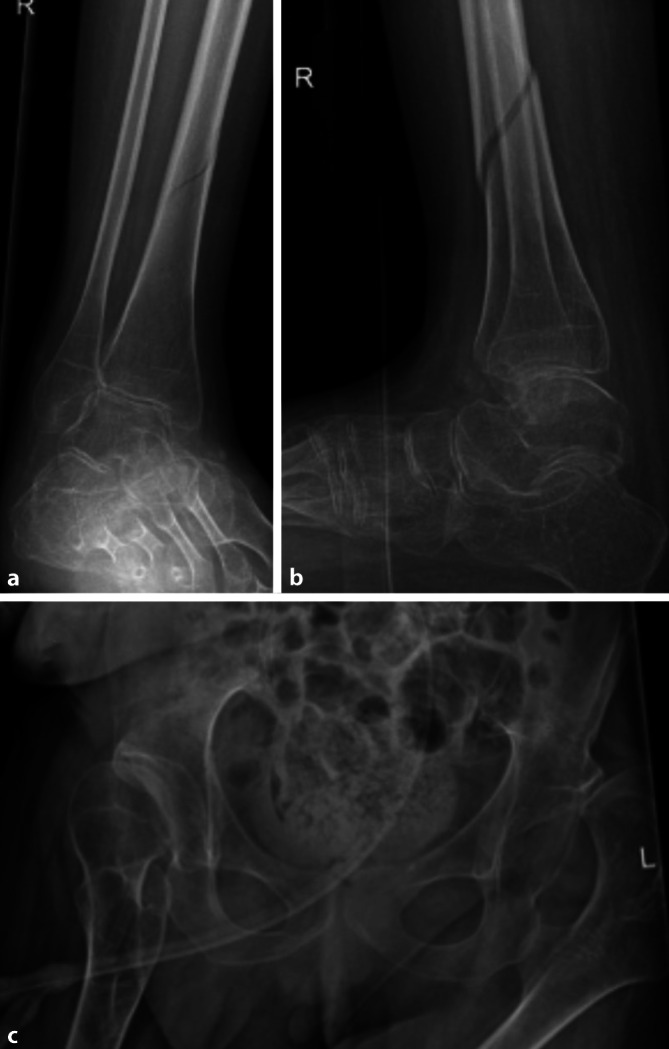

*Körperliche Behinderungen* äußern sich u. a. durch Paresen und Kontrakturen mit Einschränkungen der Gelenkbeweglichkeit bis hin zu chronischen Gelenk(sub)luxationen und hiermit verbundener reduzierter individueller Mobilität (Abb. 2). Nicht selten liegt erschwerend ein dysproportionales Knochenwachstum mit teils unphysiologischen Knochendimensionen und -achsen (z. B. geringere Markraumdurchmesser, veränderte CCD-Winkel) vor.

Zusätzlich besteht bei den meisten Patienten mit Behinderungen ein deutlich erhöhtes Risiko für Frakturen. Körperliche Beeinträchtigungen, Funktionsstörung im Bereich des Kleinhirns sowie epileptische Anfälle erhöhen die Sturzneigung [13]. Ferner können medikamentöse Kotherapien zum einen die Sturzneigung erhöhen und die Knochenqualität mindern (z. B. Antiepileptika wie Carbamazepin, Zytochrom-P450-Metabolismus) [2]. Liegt parallel eine Einschränkung der Mobilität vor, so wird die muskuloskeletale Einheit geschwächt und damit die Sturzneigung zusätzlich erhöht und parallel die Knochenqualität vermindert. Als Besonderheit sei der tonisch-klonische Anfall genannt, bei dem zusätzlich durch die Muskelkontraktion massive Kräfte auf das Skelett wirken, sodass Frakturen, Luxationen oder sogar komplexe Luxationsfrakturen auftreten können [5, 7, 14].

### Diagnostik

Die Aussagekraft der klinischen Untersuchung ist bei Menschen mit Behinderungen häufig deutlich eingeschränkt. So sind Angaben über Beschwerden, Schmerzen und Instabilitäten nicht sicher zu verwerten. Daher kommt den bildgebenden Verfahren eine entscheidende Rolle zu, um unfallchirurgische Verletzungen sicher zu identifizieren. Allerdings ist die Durchführung häufig mit zusätzlichem zeitlichen und personellen Aufwand verbunden. Vor allem Menschen mit geistigen Behinderungen können Anweisungen nicht oder nur bedingt folgen, sodass selbst die Anfertigung von konventionellen Röntgenbildern eine große Herausforderung darstellt. Die Untersuchung mittels Computertomographie (CT) oder Magnetresonanztomographie (MRT) ist häufig nur durch medikamentöse Sedierung oder in Narkose möglich. Je nach Grunderkrankung können vorliegende spastische Kontrakturen die Durchführung von nativradiologischer oder schnittbildgebender Diagnostik teils erheblich erschweren. Insbesondere die Auswertung von konventionellen Röntgenbildern kann aufgrund der fehlenden Standardeinstellungen (prä-, intra- und postoperativ) eine Herausforderung darstellen (Abb. 5f, g).

### Therapie

Die Behandlung von Frakturen bei Menschen mit Behinderungen kann nicht immer nach etablierten Konzepten folgen.

#### Konservative Therapie (unter Abwägung der primären biomechanischen Stabilität)

Unsere Erfahrungen mit diesem Patient*innenkollektiv zeigt, dass die postoperative Komplikationsrate hoch ist (z. B. Wundheilungsstörungen, Infektionen, Implantatversagen), sodass der konservativen Frakturbehandlung eine entscheidende Rolle zukommt. *Die Entscheidung zur konservativen Therapie auch bei Gelenkfrakturen muss interdisziplinär* unter Berücksichtigung des Anspruchs der Patient*innen, aber auch von Compliance und den Umständen der Versorgung getragen werden. Größenteils ist eine oft langwierige stationäre Betreuung der Patient*innen notwendig, um die Prinzipien der konservativen Therapie (Ruhigstellung, Belastungs- und Bewegungseinschränkung) suffizient umsetzen und eine entsprechende Betreuung und Pflege gewährleisten zu können.

**Figure 3:**
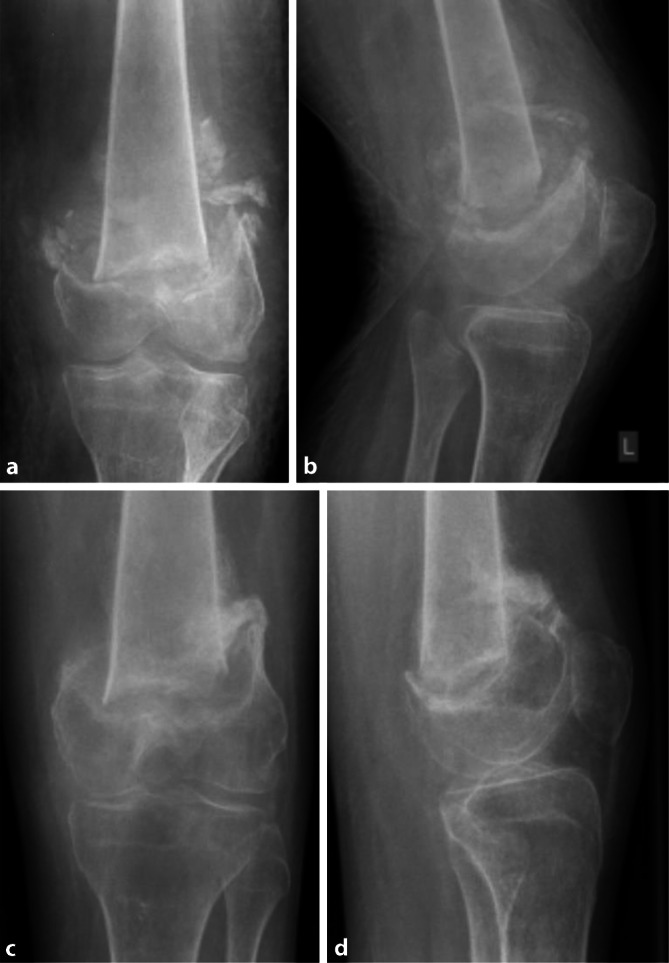

Einen Zufallsbefund zeigt Abb. 3; der 53-jährige Patient wurde bei rezidivierender Schwellung des linken Beines in unserer Ambulanz vorgestellt. Vor 3 Monaten wurde bei ihm eine Thrombose der V. femoralis diagnostiziert und mit Antikoagulanzien therapiert. Bei der erneuten Vorstellung fiel eine Beinverkürzung von ca. 5 cm auf. Der Patient leidet an einer Epilepsie mit schwerer Intelligenzminderung ohne Sprachfähigkeit sowie an einer spastischen Hemiparese. Der Patient war im Rollstuhl mobilisiert; der Transfer erfolgt mit dem Lift. Die distale Femurfraktur wurde konservativ mittels individuell angepasster spezieller Frakturorthese nach Gipsabdruck therapiert. Somit konnten die Mobilisation in den Rollstuhl sichergestellt und 9 Monate nach Therapiebeginn eine Konsolidierung erreicht werden. Entscheidend bei der Therapie von Frakturen bei Menschen mit Behinderungen ist, dass die vorhandene Mobilität durch die Therapie möglichst wenig eingeschränkt wird, um die Teilhabe auch während der Therapie zu ermöglichen.

#### Operative Therapie (unter Abwägung der primären biomechanischen Stabilität)

Die Entscheidung zur operativen Versorgung von Frakturen muss interdisziplinär und individuell getroffen werden. Besondere Berücksichtigung müssen die genannten Risikofaktoren finden. So sollten Informationen zu Sturzneigung, Verhalten, Compliance, Begleitmedikationen und Mobilität in die Entscheidung über die Indikation und das zu wählende Verfahren einfließen.

Bei der Wahl des operativen Prozederes muss das Verfahren identifiziert werden, welches eine maximale Stabilität zur Erlangung der knöchernen Konsolidierung erzielt, um die funktionellen Bedürfnisse der Patient*innen sicher wiederherzustellen. Dabei sind alternative Zugänge, Implantate, Verfahren oder auch die Kombination dieser individuell einzusetzen. Ähnlich der Alterstraumatologie schließen sich auch in der Behindertenmedizin Entlastungs- oder Teilbelastungskonzepte zumeist aus.

**Figure 4:**
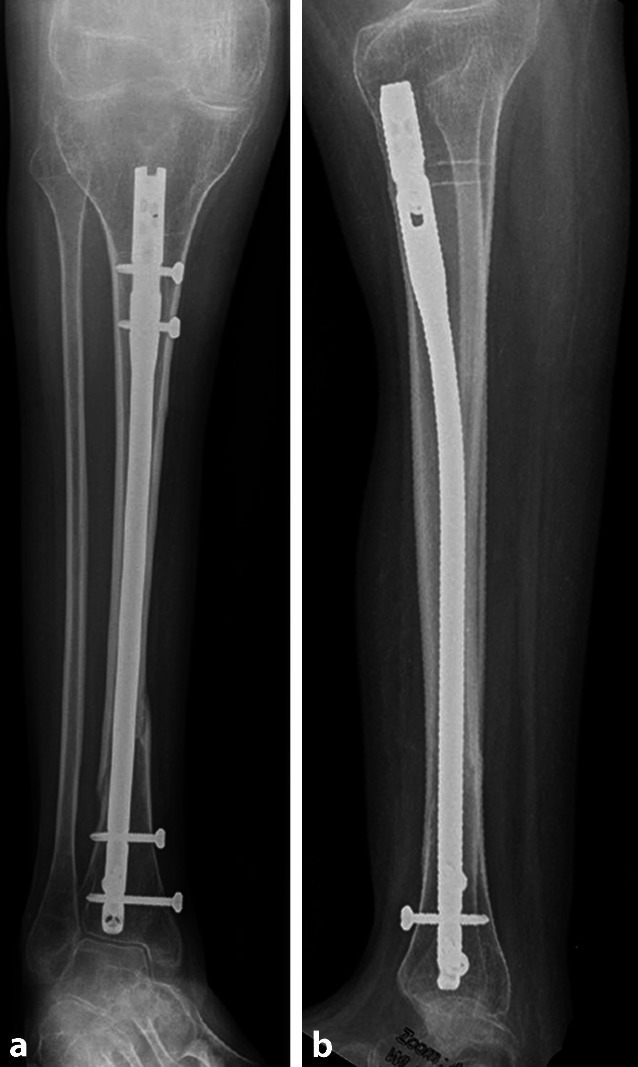

Die mittels Marknagelung versorgte distale Tibiaschaftfraktur des Patienten aus Abb. 2 mit spastischer Tetraparese mit Streckkontrakturen sämtlicher Extremitäten zeigt Abb. 4. Die Indikation zur operativen Stabilisierung wurde gestellt, um den Transfer und die Mobilisation (Rollstuhl) sicherzustellen, ausreichend Stabilität zum Schutz der Weichteile (Pat. mit Verhaltensstörungen) und Schmerzfreiheit zu erreichen. Die Versorgung erfolgte aufgrund der Kontrakturen in diesem Fall über einen suprapatellaren Zugang [3].

Zur erfolgreichen operativen Versorgung ist die präoperative Planung der Frakturversorgung in diesem Patent*innenkollektiv von entscheidender Bedeutung. Dies beinhaltet als Erstes die präoperative Überprüfung der Lagerbarkeit der Patient*innen. Kontrakturen und Luxationen können Standardlagerungen sowie gängige osteosynthetische Verfahren erheblich erschweren.

**Figure 5:**
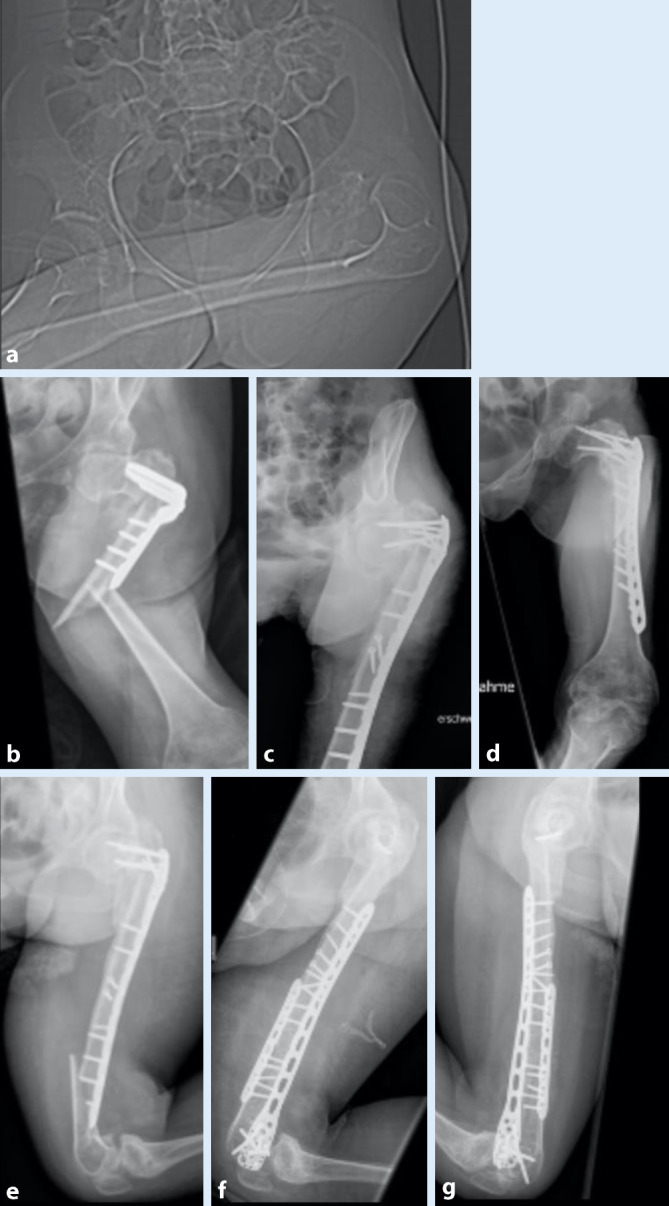

Neben Lagerung, Zugang, Reposition und Implantaten müssen auch die zu erwartenden Beanspruchungen berücksichtigt werden. So treten Belastungen auf (z. B. Transfers mittels Lift), die durch die Osteosynthese gehalten werden müssen. Zusätzlich müssen weitere Risikofaktoren, wie Osteoporose durch Immobilität und verzögerte Frakturheilung durch fehlende mechanische Stimulation, berücksichtigt werden. Den Fall einer 28-jährigen Patientin mit frühkindlichem Hirnschaden, schwerer Intelligenzminderung und einem symptomatischen Anfallsleiden zeigt Abb. 5. Zusätzlich besteht eine Tetraspastik ohne Gehfähigkeit. Die Mobilisation erfolgt im Sitzschalenrollstuhl. Bei Hüftluxation links bei Hüftdysplasie (Crowe-Typ II, Abb. 5a) mit begleitender Innenrotations- und Abduktionsbeugekontraktur erfolgte eine varisierende Umstellungsosteotomie mit einer 10-Loch-Klingenplatte (Fa. DePuy Synthes, Zuchwil, Schweiz). 3 Monate postoperativ fiel bei der Mobilisation in den Rollstuhl eine abnorme Beweglichkeit des linken Oberschenkels auf. Die durchgeführte Röntgendiagnostik zeigte eine periimplantäre Femurfraktur (Abb. 5b), welche aufgrund der anatomischen Verhältnisse mit einer proximalen Humerusplatte (Philos, Fa. DePuy Synthes, Zuchwil, Schweiz) versorgt wurde (Abb. 5c, d). Wiederum 9 Monate später kam es zu einer erneuten periimplantären distalen Femurfraktur (Abb. 5e). Diese wurde mittels Doppelplattenosteosynthese stabilisiert, dazu wurde eine lange Philos-Platte (Fa. DePuy Synthes, Zuchwil, Schweiz) mit einer ventralen „Locking-compression“-Platte (Fa. DePuy Synthes, Zuchwil, Schweiz; Abb. 5f, g) kombiniert.

**Figure 6:**
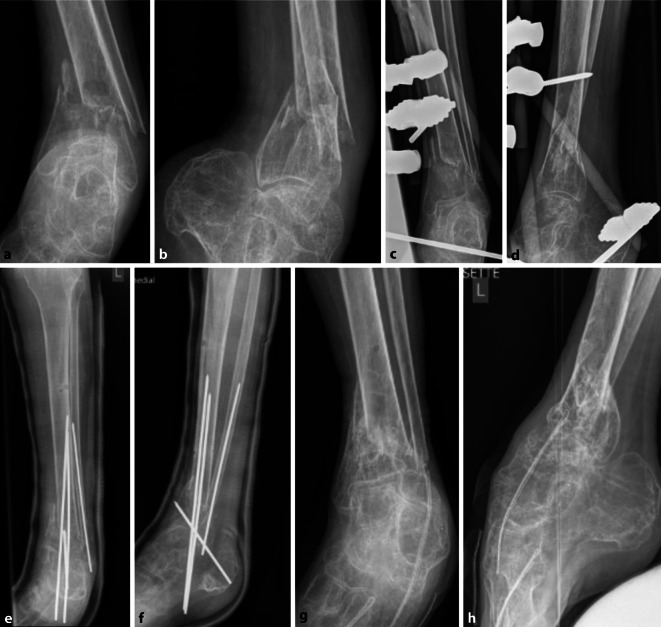

Bei der operativen Therapie von Frakturen bei Menschen mit Behinderungen sind die Ziele der Arbeitsgemeinschaft für Osteosynthesefragen (AO) nicht immer vollständig zu erreichen. So sind für jeden Patienten die Ziele individuell festzulegen. Für die in Abb. 6a, b gezeigte distale Unterschenkelfraktur einer 63-jährigen Patientin galt es, die knöcherne Konsolidierung mit minimal-invasiven Methoden (Weichteilprobleme) zu erreichen. Das Erzielen einer anatomischen Stellung bei vorbestehender ausgeprägter Spitzfußstellung war aufgrund der spastischen Tetraparese mit Kontrakturen sowie der weiteren Begleiterkrankungen (Epilepsie sowie schwere kognitive Beeinträchtigungen und Wesensveränderungen) nicht primäres Ziel der Therapie. Die initiale Stabilisierung erfolgte durch die Anlage eines sprunggelenkübergreifenden Fixateur externe (Abb. 6c, d). Nach 5 Wochen erfolgten der Wechsel auf eine intramedulläre Schienung mittels K‑Drähten und die Anlage einer additiven Gipsschiene (Abb. 6e, f). Im Verlauf erfolgte die sukzessive Entfernung der K‑Drähte; nach 9 Monaten war die knöcherne Konsolidierung erreicht (Abb. 6g, h).

**Figure 7:**
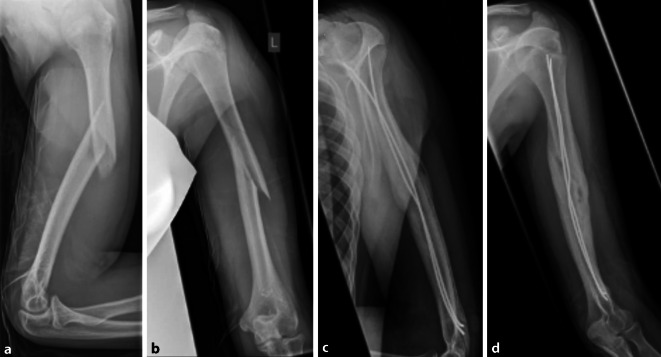

Die Wahl des operativen Verfahrens, der Implantate und das geplante Nachbehandlungsschema haben entscheidende Auswirkungen auf den Alltag und die Teilhabe des beschriebenen Patient*innenkollektives. Die Humerusschaftfraktur (AO 12-C2) eines 16-jährigen Patienten mit psychomotorischer Entwicklungsstörung nach Frühgeburtlichkeit, beinbetonter bilateraler spastischer Zerebralparese und Epilepsie zeigt Abb. 7a, b. Diese wurde in ESIN-Technik (ESIN: elastisch stabile intramedulläre Nagelung) versorgt (Abb. 7c, d), dabei handelt es sich um eine nichtbelastungsstabile Osteosynthese. Aufgrund der nötigen Entlastung des linken Arms erfolgte die Mobilisation des Patienten lediglich im Rollstuhl. Dadurch und durch eine sukzessiv entstandene Schonhaltung des linken Armes war der Patient ca. 2 Jahre lang nicht mobilisierbar. Vor der Fraktur war der Patient mit Hilfestellung einer Person bzw. unter Aufsicht in der Lage, kurze Wege zu Fuß zurückzulegen. Trotz den Alters wäre für diesen Patienten eine stabile Osteosynthese mittels Platte oder Marknagel vorteilhafter gewesen.

Die verwendeten Implantate sind selbstverständlich individuell zu wählen. In Abhängigkeit von der Frakturmorphologie sind Verriegelungsmarknägel mit großem Durchmesser bei diaphysären Verletzungen oder möglichst großvolumige Plattensysteme mit Option zur winkelstabilen Schraubenverankerung im metadiaphysären Übergang geeignete Verfahren. Dies kann Komplikationen durch Non-Compliance, verminderte Knochenqualität oder der Kombination verhindern.

**Figure 8:**
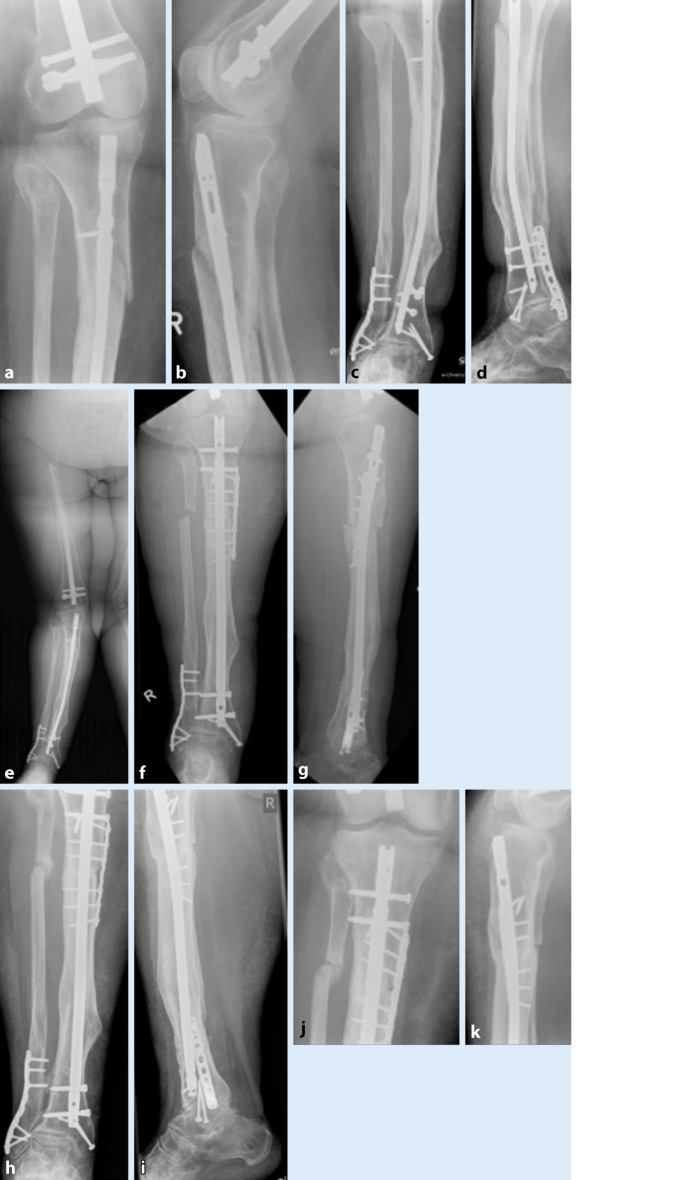

Da es sich um ein Patientenkollektiv mit hohem Frakturrisiko handelt, muss bei jeder Versorgung auf die Wiederherstellung der Achse geachtet werden, um für eine kompensierte Biomechanik zu sorgen und so Komplikationen zu vermeiden. In Abb. 8 ist der Fall einer 27-jährigen Patientin gezeigt, die bei liegendem Tibia- und Femurnagel (Z. n. distaler Unterschenkelfraktur und Oberschenkelfraktur) eine proximale Unterschenkelfraktur (Abb. 8a–d) mit Deformierung des einliegenden Implantats erlitten hat (Abb. 8a–e). Die Patientin leidet an einer Epilepsie mit mittelgradiger Intelligenzminderung sowie einer Adipositas III°. Der initial konservative Therapieversuch mit Belassen des einliegenden deformierten Nagels führte innerhalb von 6 Monaten nicht zu einer knöchernen Durchbauung der Fraktur. Die daraufhin durchgeführte Operation mit Korrektur der Achse und Erhöhung des Marknageldurchmessers und additiver Plattenosteosynthese (Abb. 8f, g) resultierte in einer knöchernen Konsolidierung der Fraktur (Abb. 8h–k).

Auch die Doppelplattenosteosynthese, z. B. bei erheblich reduzierter Knochenqualität oder durch die Grunderkrankung bedingter sehr hoher Incompliance, stellt eine mögliche Technik dar, die in der Lage ist, die Primärstabilität deutlich zu erhöhen (Abb. 5; [6, 8, 9, 11]).

Bei der Anlage der Wundverbände ist eine reduzierte postoperative Compliance zu antizipieren; Material und Technik sind so zu wählen, dass ein Belassen des Verbands für mehrere Tage möglich ist. In seltenen Fällen (z. B. Gelenkersatz nach Schenkelhalsfraktur, Osteosynthesen an langen Röhrenknochen) eignet sich die Unterdruckwundtherapie (NPWT) mit Verbandtragedauern bis zu 7 Tagen, wobei in der täglichen Versorgung auf die anhängende Pumpe zu achten ist. Bei der Verwendung von additiven immobilisierenden Schienen sollte auf eine suffiziente Polsterung geachtet werden, um Druckstellen und Ulzera zu vermeiden.

## Nachbehandlung

Die postoperative Therapie bei Menschen mit einer Behinderung gestaltet sich oft sehr schwierig und ist meist unter stationären Bedingungen durchzuführen.

Unmittelbar nach dem operativen Eingriff kann nur bei geeigneten Patienten mit suffizienter Zugänglichkeit und entsprechender Compliance eine direkte Nachbehandlung auf der Normalstation durchgeführt werden. Ein Großteil der Patienten muss zumindest kurzfristig observations- oder intensivstationär behandelt werden.

Hinsichtlich der fachspezifisch unfallchirurgischen Nachbehandlung sind etablierte Schemata zu modifizieren. Teilbelastungen können regelhaft nicht eingehalten werden. Auch ist in Fällen akuter Agitiertheit zu prüfen, ob die Osteosynthese durch zusätzliche Schienung, Bracing oder Gips geschützt werden muss. Manipulationen an Verbänden und Wunden durch den Patienten sind ein wichtiger Risikofaktor, der bedacht und adressiert werden muss. Hier bieten sich geschlossene Systeme zum sicheren Wundverschluss (PICO VAC sure wound closure nach WHO, Smith & Nephew, Hamburg, Deutschland) oder aber auch zirkuläre Schienenverbände bis zum Abschluss der Wundheilung an.

Die Einleitung einer Abklärung und ggf. Therapie bei Verdacht auf Osteoporose ist im Sinne der Frakturprophylaxe sinnvoll [12].

Aufgrund des deutlich erhöhten Frakturrisikos im beschriebenen Patientenkollektiv muss über die Entfernung der eingebrachten Implantate kritisch nachgedacht werden, um ggf. komplizierte periimplantäre Folgefrakturen zu vermeiden.

## Fazit für die Praxis

Die Behandlung von Verletzungen bei Menschen mit schweren geistigen und körperlichen Behinderungen stellt eine große Herausforderung dar und kann nur im interdisziplinären Ansatz erfolgreich durchgeführt werden. Die Ansprüche an die konservative wie auch operative Unfallchirurgie ergeben sich in diesem Patientenkollektiv aus den individuellen Ansprüchen der Patient*innen, aus der Abwägung zu erwartender Komplikationen und Risiken und der Anwendung auch im Spiegel der geltenden AO-Standards oft unkonventioneller Therapiekombinationen. Dabei ist die Behandlung im stationären Aufenthalt langwierig, personalintensiv und kann häufig nur in spezialisierten Einrichtungen mit speziell ausgebildetem Pflegenden und Therapeuten ermöglicht werden.
